# The prevalence of zinc deficiency in morbidly obese patients before and after different types of bariatric surgery

**DOI:** 10.1186/s12902-021-00763-0

**Published:** 2021-05-25

**Authors:** Fahimeh Soheilipour, Mohammad Ebrahimian, Mohadeseh Pishgahroudsari, Maryam Hajian, Davoud Amirkashani, Mahtab Ordooei, Mohammad Radgoodarzi, Delaram Eskandari

**Affiliations:** 1 Minimally Invasive Surgery Research Center, Iran University of Medical Sciences, Tehran, Iran; 2 Faculty of Medicine, Iran University of Medical Sciences, Tehran, Iran; 3 Pediatric Endocrinology Department, Ali Asghar Children Hospital, Iran University of Medical Sciences, Tehran, Iran; 4 Children Growth Disorder Research Center, Shahid Sadoughi University of Medical Sciences, Yazd, Iran; 5 Endocrinology Department, Rasool Akram Medical Complex, Iran University of Medical Sciences, Tehran, Iran

**Keywords:** Obesity, Bariatric surgery, Morbid

## Abstract

**Background:**

The prevalence of obesity is considered to be increased worldwide. Lack of mineral elements is one of the essential side effects of bariatric surgery as a trending treatment for obesity. We aimed to assess zinc deficiency among morbidly obese patients before and following different types of bariatric surgical procedures.

**Methods:**

In the present retrospective cohort study, 413 morbidly obese patients (body mass index (BMI) ≥ 40 kg/m2 or BMI ≥ 35 kg/m2 with a complication or risk factor, e.g., diabetes mellitus) were enrolled who received bariatric surgery, aged between 18 and 65 years old, and had a negative history of active consumption of alcohol and illicit drugs. Patients were assigned into three groups of bariatric surgeries: mini-gastric bypass, Roux-en-Y gastric bypass (RYGB), and sleeve gastrectomy (SG). We recorded baseline clinical and demographic characteristics and zinc serum levels during the preoperative and postoperative follow-up periods at three, six, and 12 months after the operation.

**Results:**

All patients with a mean age of 40.57 ± 10.63 years and a mean preoperative BMI of 45.78 ± 6.02 kg/m2 underwent bariatric surgery. 10.2% of the bariatric patients experienced zinc deficiency before the surgery, and 27.1% at 1 year after the surgery. The results showed that 27.7% of mini-gastric bypass patients, 29.8% of RYGB, and 13.3% of SG experienced zinc deficiency 12 months following surgery. We observed no statistical differences in the preoperative and postoperative zinc deficiency between different types of surgeries.

**Conclusion:**

A high prevalence of preoperative zinc deficiency among morbidly obese patients who underwent bariatric surgery was observed, which increased during the postoperative periods. We recommend assessing zinc serum levels and prescribing zinc supplements before the bariatric operation to alleviate the prevalence of zinc deficiency after the operation.

**Supplementary Information:**

The online version contains supplementary material available at 10.1186/s12902-021-00763-0.

## Introduction

Obesity, as one of the most crucial health issues worldwide, is considered a modifiable and preventable risk factor for many diseases. The increased rate of obesity raises the mortality and morbidity rates in populations. The main consequences are cardiovascular diseases, cerebrovascular accidents and strokes,, and cancer types [1]. Approximately 600 million people are obese globally, and the prevalence of obesity in Iran was reported by 21.4% in 2019 [2]. Bariatric surgery is a trend for obesity treatment recently. Several types of bariatric operationsare classified into restrictive procedures, including sleeve gastrectomy (SG), malabsorptive procedures, such as biliopancreatic diversion with or without a duodenal switch, and combined procedures, like Roux-en-Y gastric bypass (RYGB) and mini-gastric bypass [3].

The trace elements, including zinc, copper, selenium, and iron, playan essential role in biomedical pathways for cell function and metabolism in cells [4]. Zinc is a catalytic component of many metalloenzymesand transcription factors found in all body tissues, fluids, and secretions [5]. The zinc serum levels are reduced in obesity, indicating the correlation of zinc deficiency and obesity-related complications such as insulin resistance [6]. The serum zinc levels are the main pool of available zinc in the human body. Therefore, zinc deficiency could affect zinc hemostasis [7]. The zinc deficiency can cause hair loss, diarrhea, glossitis, nail dystrophy, hypogonadisms in males, infertility, and anemia, delayed wound healing, skin lesion, and taste alteration [8]. The lack of micronutrients reported in most studies is one of the common complications after bariatric surgery. One of them is the four-year follow-up of 65 obese patients after bariatric surgery that showed basal deficiencies in obese patients, which is increased after bariatric surgery [9].

This study aimed to assess the zinc deficiency in morbidly obese patients after bariatric surgeries and its changes according to the types of surgical procedures among the Iranian population.

## Main text

### Methods

We conducted a retrospective cohort study at the obesity center of RasoulAkramHospital, Tehran, Iran, fromDecember2018 to December2019. We recruited 413 morbidly obese patients (body mass index (BMI) ≥ 40 kg/m^2^ or BMI ≥ 35 kg/m^2^ with a complication or risk factor, e.g., DM) who underwent bariatric surgery, aged between 18 and 65 years old, and did not actively consume alcohol and illicit drugs. Patients who had a history of bariatric surgery were excluded. The Ethics Committee of Iran University of Medical Sciences approved the present study (Ethics code: IR.IUMS.REC 1396.31866).

We assigned the patients into three groups according to the type of bariatric surgery (mini-gastric bypass, RYGB, and SG). We recorded zinc serum levels during the preoperative period and follow-up measurements at three, six, and 12 months after the surgery. The reference value for zinc serum levels is 70-120 μg/dl [10]. Baseline clinical and demographic features were also recorded on a checklist.

At the first postoperative visit (2–3 weeks after the operation), we encouraged the patients to take multivitamin and mineral supplementations postoperatively (one tablet orally daily), which contained30 mg zinc as zinc oxide per tablet alongside other vitamins and micronutrients, presented in Table 1.

**Table 1:** Ingredients of multivitamin and mineral supplementation

Nutrient	Amount per tablet
**Vitamin A (as Retinyl Acetate and Beta Carotene)**	2250 μg
**Vitamin C (Ascorbic acid)**	90 mg
**Vitamin D (as Cholecalciferol)**	75 μg
**Vitamin E (as D-Alpha Tocopheryl Succinate)**	40.2 μg
**Vitamin K (as Phytonadione)**	160 μg
**Thiamin (as Thiamin HCl)**	3 mg
**Riboflavin**	3.4 mg
**Niacin (as Niacinamide)**	20 mg
**Vitamin B6 (as Pyridoxine HCl)**	4 mg
**Folate (as Folic Acid)**	800 μg
**Vitamin B12 (as Cyanocobalamin)**	500 μg
**Biotin**	600 μg
**Pantothenic acid (as Calcium D-Pantothenate)**	20 mg
**Iron (as Ferrous Fumarate)**	45 mg
**Iodine (as Potassium Iodide)**	150 μg
**Magnesium (as Magnesium Oxide)**	400 mg
**Zinc (as Zinc Oxide)**	30 mg
**Selenium (as L-Selenomethionine)**	70 μg
**Copper (as Cupric Oxide)**	2 mg
**Manganese (as Manganese Sulfate)**	2 mg
**Chromium (as Chromium Picolinate)**	120 μg
**Molybdenum (as Sodium Molybdate)**	75 μg

#### Statistical analysis

We performed statistical analyses using IBM SPSS statistics version 19. Continuous variables were demonstrated as mean ± standard deviation (SD) or median and categorical values as percentages or absolute values. To compare continuous variables, we used ANOVA or Kruskal-Wallis test based on the assumptions of the former. In case the ANOVA or Kruskal-Wallis test met the significant levels, we performed Tukey post hoc test. We made a comparison of categorical values by Chi-square or Fisher’s exact tests. We considered 2-tailed *p* values of < 0.05 statistically significant.

## Results

### Demographics

Four hundred thirteen (413) patients met the inclusion criteria, and their demographic information is shown in Table 2. In Total, all patients with a mean age of 40.57 ± 10.63 years and a mean preoperative BMI of 45.78 ± 6.02 kg/m^2^underwent bariatric surgery. Significantly more patients were female (334, 83.3%), and more underwent mini-gastric bypass than RYGB or SG (289, 94, and 30, respectively). The ANOVA test for age between surgical groups was not significant (*P*-value: 0.413). The frequency of comorbidities, including hypertension, DM type II, hyperlipidemia, and hypothyroidism, were summarized in Table 1. Hyperlipidemia was the most common comorbidity in all patients (188/413).

**Table 2:** Demographic and clinical characteristics

Characteristic	
Age (years), mean (SD)	40.57 (10.63)
Sex, female, n (%)	344 (83.3)
Preoperative BMI (kg/m^2^), mean (SD)	45.78 (6.02)
Type of bariatric surgery, n (%)	
Mini-gastric bypass	289 (70)
RYGB	94 (22.8)
SG	30 (7.2)
HTN, n (%)	79 (19.1)
Impaired FBS, n (%)	76 (18.4)
DM type II, n (%)	76 (18.4)
Dyslipidemia, n (%)	188 (45.5)
Hypothyroidism, n (%)	91 (22)

*Abbreviations*: *BMI* Body mass index, *RYGB* Roux-en-Y gastric bypass, *SG* Sleeve gastrectomy, *HTN* Hypertension, *FBS* Fasting blood sugar, *DM* Diabetes mellitus, *SD* Standard deviation

### Zinc serum levels and zinc deficiency

The mean preoperativezinc plasma concentration was 89.96 ± 21.4 μg/dl. Thezincserum level after surgery was significantly decreased in patients (*P*-value< 0.001), with statistically significant differences between the preoperative period and the six and 12 months after the operation, as well as between the third postoperative month and the six and 12 months after the operation. There was an association between BMI groups and zinc serum levels (P-value: 0.044), with a statistical difference between 40 ≤ BMI ≤ 50 and BMI > 50 groups. Furthermore, the association between sex and zinc serum levels was significant (P-value: 0.038). The data weresummarized in Table 3. There was no association between zinc serum levels and any comorbidities. However,there was a significant association between impaired FBS, DM type II, and zinc deficiency (P-value: 0.027, and 0.047, respectively). The zinc plasma concentration was shown in Table S1 (Supplementary file: Tables) based on the type of bariatric procedures. At 12 months of surgery, the zinc serum level presented significantly lower in the mini-gastric bypass compared to the other types of surgeries. We compared zinc deficiency between groups of bariatric surgery before and after the operation, shown in Table S2 (Supplementary file: Tables)). The results showed that at 12 months after operation, 27.7% of the patients who underwent mini-gastric bypass, 29.8% of RYGB, and 13.3% of SG experienced zinc deficiency. However, no significant difference in zinc deficiency between different types of surgery was observed.

**Table 3:** Zinc serum level and zinc deficiency during the pre- and postoperative period and in different groups of BMI, sex, and underlying diseases

	Zinc serum level (μg/dl), mean (SD)	*P*-value	Zinc deficiency, n (%)	*P*-value
Preoperative	89.96 (21.04) ^a^	< 0.001	42 (10.2)	–
At 3 months	89.99 (26.35) ^b^		67 (16.2)	
At 6 months	79.16 (18.39) ^a b^		119 (28.8)	
At 12 months	80.07 (17.92) ^a b^		112 (27.1)	
Group variables				
BMI		0.044		0.372
35 ≤ BMI < 40 (*n* = 44)	95.79 (33.92)		4 (9.1)	
40 ≤ BMI ≤ 50 (*n* = 277)	87.97 (18.06)^a^		32 (11.6)	
BMI > 50 (*n* = 92)	93.16 (20.77)^a^		6 (6.5)	
Sex		0.038		0.029
Female (*n* = 344)	89.43 (21.99)		40 (11.6)	
Male (*n* = 69)	92.58 (15.28)		2 (2.9)	
HTN		0.210		0.689
Yes (*n* = 79)	87.37 (18.11)		9 (11.4)	
No (*n* = 334)	90.57 (21.65)		33 (9.9)	
Impaired FBS		0.227		0.027
Yes (*n* = 76)	87.70 (22.12)		13 (17.1)	
No (*n* = 337)	90.47 (20.78)		29 (8.6)	
DM Type II		0.503		0.047
Yes (*n* = 76)	92.25 (26.10)		3 (3.9)	
No (*n* = 337)	89.44 (19.73)		39 (11.6)	
Hyperlipidemia		0.584		0.308
Yes (*n* = 188)	90.78 (22.50)		16 (8.5)	
No (*n* = 225)	89.27 (19.75)		26 (11.6)	
Hypothyroidism		0.861		0.376
Yes (*n* = 91)	90.42 (20.95)		7 (7.7)	
No (*n* = 322)	89.83 (21.09)		35 (10.9)	

*Abbreviations*: *BMI* Body mass index, *HTN* Hypertension, *FBS* Fasting blood sugar, *DM* Diabetes mellitus, *SD* Standard deviation

^a^, and ^b^ indicate differences between groups

## Discussion

In the current study, we demonstrated that bariatric patients tended to show a high proportion of zinc deficiency increased from 10.2% in the preoperative period to 27.1% at 1 year after the surgery. There are reports which presented a high prevalence of zinc deficiency among obese patients during the preoperative period [11–15]. Previous studies have represented the following reasons for explaining zinc deficiency in obese patients: 1. The chronic inflammation in obese patients promotes metallothionein and zinc-copper transporter expression, resulting in the accumulation of the metal in hepatocytes and adipocytes, responsible for decreased zinc serum levels [16], 2. Inadequate intake of zinc resources with high concentrations of this micronutrient might account for zinc deficiency [17].

Furthermore, the prevalence of zinc deficiency is considered to be increased following bariatric surgery [13]. It has been revealed that 4–9% of bariatric patients experience zinc deficiency preoperatively and 20–24% after 18 months of bariatric operation [18–22]. A small-sized randomized controlled trial of severe and morbidly obese female patients who underwent RYGB reported a significant decrease in zinc absorption from 32.3 to 13.6% after 6 months of RYGB [20]. Decreased zinc absorption has been described by reducing the acidic environment following RYGB and SG [23].

However, Rojas et al. noted that zinc plasma levels seemed to increase at 6 months after RYGB among severe or morbidly obese women. They attributed such an increase in zinc plasma levels to reducing inflammation presented in their patients after the surgery. Nonetheless, they mentioned the “size of the exchangeable zinc pool” as a suitable zinc status parameter, which significantly decreased after RYGB [23].

We have shown that preoperative zinc serum levels were significantly lower among bariatric patients with 40 ≤ BMI < 50 kg/m^2^, but we did not find an association between zinc deficiency and BMI. Consistently, Mohammadi et al. presented that morbidly obese patients with BMI > 50 kg/m^2^have lower zinc serum levels while there was no relation between zinc deficiency and an increasing BMI [15]. However, it has been reported that zinc serum levels before bariatric surgery seem to be not related to BMI [13, 24, 25]. Differences in:1) Cultural and geographical factors, which could affect zinc deficiency, 2) study designs, 3) Data distribution in BMI groups, and 4. dietary intake or nutritional habits might potentially account for such contrast findings between reports [15].

In our study, significantly lower serum zinc concentrations and higher proportions of zinc deficiency were observed in female patients before the surgery. Compared to the male gender, higher rates of zinc deficiency in females could be attributed to different food patterns. Our findings were in agreement with the Mohammadi et al. study, which reported a similar pattern of zinc deficiency in women patients while they found no significant association between zinc deficiency and sex [15].

Comparing zinc deficiency among different surgical techniques, we found that 27.7% of the patients who received mini-gastric bypass, 29.8% of RYGB, and 13.3% of SG experienced zinc deficiency 12 months after surgery. It can be also interpreted that zinc deficiency increased 18% among mini-gastric bypass and 19.2% among RYGB patients from the preoperative period to 12 months after the surgery. However, no significant difference was observed in zinc deficiency between groups of bariatric surgery. Similar to our findings, Ferraz et al. observed no significant difference in zinc deficiency between RYGB and SG after 12 months of surgery (25.6% vs. 26.6%, respectively). Nevertheless, they presented a substantial difference between the surgeries at 24 months of follow-up (RYGB: 30% vs. SG: 6.6%, *P*-value< 0.05) [10]. Consistently, a higher prevalence of zinc deficiency among RYGB patients compared to SG patients following 12 months of operation has been reported (40.7% vs. 18.8%, respectively) [13]. Concerning such a significant difference, some explanations have been offered: 1. RYGB results in food transit deviation, 2. SG optimizes gastric and proximal intestinal emptying time, which helps the nutrients maintain more prolonged contact with the jejunum and proximal ileum and leads to a better adhesion to vitamin supplementation [10].

It has been reported that zinc deficiency could cause different disorders, including skin disorders (e.g. bullous dermatitis, psoriasiform and eczematous lesions), and be fatal in severe cases [5, 26, 27]. In addition, the deficiency of this micronutrient might contribute to poor recovery during the postoperative period [15], so preoperative baseline assessment and correction of zinc deficiency could improve its prevalence in the postoperative period. Previous studies suggested that zinc deficiency might be prevented by administration of zinc supplementations after bariatric surgery [19]. American Association of Clinical Endocrinologists (AACE) guidelines has recommended taking a daily multivitamin supplementation following obesity surgery to achieve dietary reference intakes of zinc, which equal 8–11 mg per day [28]. Despite the fact that our multivitamin and mineral supplementations contained 30 mg of elemental zinc, we observed a high prevalence of zinc deficiency among our patients at the end of the study period. However, the average zinc serum levels were within the normal range in our patients during the postoperative period.

Collectively, in the present cohort study, we assessed the zinc status in a population of morbidly obese patients during the pre- and post-bariatric periods and compared zinc deficiency between different groups of bariatric surgeries within the first year of operation. We observed a high prevalence of preoperative zinc deficiency among morbidly obese patients who underwent bariatric surgery, which increased during the postoperative period. Besides, we found that mini-gastric bypass and RYGB, compared to SG, showed higher zinc deficiency rates at the end of the 12-month follow-up. However, we recommend an assessment of zinc serum levels and prescribing zinc supplementation before bariatric surgery to alleviate the prevalence of zinc deficiency after the operation.

### Strengths and limitations

Our study’s strengths included: 1. cohort study design and the large study population, 2. we compared different bariatric operations regarding zinc deficiency during the 12 months of follow-up that helped us extend the currently available data concerning zinc status in different bariatric surgical procedures. However, we encountered some limitations. First, we were unsure of whether patients take their multivitamin and mineral supplementations on a daily and regular basis after the surgery. Second, we did not evaluate confounding variables, including albumin and C-reactive protein (CRP) serum levels, which might affect the status of trace elements. Third, different distribution of patients among bariatric surgery categories, as well as the small number of patients in SG group, which could explain no significant difference in zinc deficiency between bariatric surgery groups. Forth, some modifiable variables might change the zinc serum levels, including dietary intakes and food habits. Fifth, patients’ compliance with the multivitamin and mineral supplementations following the operation was not recorded, which could possibly have an impact on zinc serum levels.

To track the exact changes in the prevalence of zinc deficiency among different types of bariatric surgeries during the pre- and postoperative period, we suggest further prospective studies with long-term follow-ups, assigning a similar number of patients into each group of surgery and matching study participants regarding nutritional habits, monitoring the patients to receive their multivitamin and micronutrient supplementations regularly after the operation, and evaluating the albumin and CRP serum levels as potential confounders.

## Supplementary Information

**Additional file 1: Table S1**. Zinc serum levels among bariatric surgery groups during the pre- and postoperative periods. **Table S2**. Zinc deficiency among bariatric surgery groups during the pre- and postoperative periods.

## Data Availability

The authors agree with sharing, coping, and modifying the data used in this article, even for commercial purposes. The datasets used and analysed during the current study are available from the corresponding author on reasonable request.

## Abbreviations

BMI: Body Mass Index
RYGB: Roux-en-Y Gastric Bypass
SG: Sleeve Gastrectomy
DM: Diabetes mellitus
CRP: C-Reactive Protein
