# Supplementary material for: The prevalence of zinc deficiency in morbidly obese patients before and after different types of bariatric surgery
Source: BMC Endocr Disord. 2021 May 25;21:107. doi: 10.1186/s12902-021-00763-0 (PMC8147400; doi:10.1186/s12902-021-00763-0)
Supplement: Supplementary file 1 — Additional file 1: Table S1. Zinc serum levels among bariatric surgery groups during the pre- and postoperative periods. Table S2. Zinc deficiency among bariatric surgery groups during the pre- and postoperative periods. [file 12902_2021_763_MOESM1_ESM.docx]

**Table S1**. Zinc serum levels among bariatric surgery groups during the pre- and postoperative periods

|  | Preoperative | At 3 months | At 6 months | At 12 months |
| --- | --- | --- | --- | --- |
| Mini-gastric bypass (n=289), (µg/dl) | 90.13 (20.93) | 89.64 (26.98) | 78.30 (18.22) | 79.04 (17.47) * |
| RYGB  (n=94), (µg/dl) | 91.17 (22.14) | 92.17 (25.33) | 80.61 (19.59) | 81.73 (20.17) |
| SG  (n=30), (µg/dl) | 84.50 (18.14) | 86.56 (23.47) | 82.88 (15.71) | 84.85 (13.70) * |
| P-value | 0.096^1^ | 0.818^1^ | 0.296^2^ | 0.034^1^ |

The mean (SD) is reported.

Abbreviations: RYGB: Roux-en-Y gastric bypass, SG: Sleeve gastrectomy.

^1^Kruskal Wallis Test, ^2^ANOVA.

* indicate differences between groups.

**Table S2.**Prevalence of zinc deficiencyamong bariatric surgery groups during the pre- and postoperative periods

|  | Preoperative | At 3 months | At 6 months | At 12 months | P-value |
| --- | --- | --- | --- | --- | --- |
| Mini-gastric bypass (n=289) | 28 (9.7%) * | 45 (15.6%) * ¶ | 92 (31.8%) * ¶ | 80 (27.7%) * ¶ | <0.001 |
| RYGB (n=94) | 10 (10.6%) * | 17 (18.1%) | 22 (23.4%) * | 28 (29.8%) * | 0.004 |
| SG (n=30) | 4 (13.3%) | 5 (16.7%) | 5 (16.7%) | 4 (13.3%) | 0.967 |
| P-value | 0.809 | 0.846 | 0.091 | 0.195 |  |

The frequency and percentage were reported.

Abbreviations: RYGB: Roux-en-Y gastric bypass, SG: Sleeve gastrectomy.

*, and ¶ indicate differences between groups.
